# Differences in Muscle Inter-Phasic Coherence During Side Kick Techniques Among Male Sanda Athletes of Different Skill Levels Based on Sensor Analysis: A Cross-Sectional Study

**DOI:** 10.3390/s26020646

**Published:** 2026-01-18

**Authors:** Liang Li, Tianxing Liu, Guixian Wang

**Affiliations:** 1 China Wushu School, Beijing Sport University, Beijing 100084, China; 15311129560@163.com (L.L.); 15136286681@163.com (T.L.); 2 School of General Education, Beijing Normal-Hong Kong Baptist University, Zhuhai 519017, China

**Keywords:** sanda athletes, side kick technique, intermuscular coherence, surface electromyography (sEMG), skill level differentiation

## Abstract

**Objective:** to clarify differences in the intermuscular coherence of core muscles during side kicks among male Sanda athletes at varying skill levels, particularly in critical frequency bands; to reveal the association between neuromuscular coordination mechanisms and technical proficiency; and to provide methodological references for quantitative analysis of combat sports techniques. **Methods:** Thirty-six male Sanda athletes were divided into professional (*n* = 18) and amateur (*n* = 18) groups based on athletic ranking and training duration. Surface electromyographic (EMG) signals from 15 core muscles and kinematic data were synchronously recorded using a wireless EMG system and a high-speed camera. Signal processing extracted root mean square amplitude (RMS) and integral EMG (iEMG). Muscle coordination was quantified via time-frequency coherence analysis across alpha (8–15 Hz), beta (15–30 Hz), and gamma (30–50 Hz) bands. **Results:** The professional group exhibited significantly higher RMS and iEMG values in most core muscles (e.g., rectus femoris RMS: 0.298 ± 0.072 vs. 0.214 ± 0.077 mV, *p* < 0.001). Regarding intermuscular coherence, the professional group demonstrated significantly superior coherence in the α, β, and γ bands for key muscle pairs, including upper limb–swing leg, support leg–swing leg, and upper limb–support leg. Notable differences were observed in pairs such as external oblique–rectus femoris (alpha band: 0.039 ± 0.012 vs. 0.032 ± 0.011, *p* < 0.01) and right rectus femoris–biceps femoris (beta band: 0.033 ± 0.010 vs. 0.023 ± 0.007, *p* < 0.01). **Conclusions:** The fundamental difference in side kick technique among Sanda athletes lies in neuromuscular control strategies and muscle coordination efficiency. Sensor-based intermuscular coherence analysis provides an objective quantitative indicator for distinguishing technical proficiency, offering a scientific basis for optimizing training and extending the methodological framework for technique assessment in combat sports.

## 1. Introduction

As a combat sport characterized by direct confrontation, the technical proficiency in Sanda directly determines the outcome of competition. Its core lies in the coordinated manifestation of movement power, speed, accuracy, and stability [1]. Traditional training models relying solely on subjective evaluation can no longer meet the demand for precise improvement. There is an urgent need to introduce objective biomechanical and neuromuscular control metrics to achieve quantitative assessment of specialized technical quality. Surface electromyography (sEMG) signals, as a core tool reflecting neuromuscular coordination, are widely applied in movement technique analysis, providing scientific evidence for revealing technical differences among athletes of varying skill levels [2,3].

Sanda, also known as Chinese Combat, is a modern full-contact competitive combat sport originating from Chinese martial arts. Its technical system highly integrates punching, kicking, and grappling techniques, emphasizing the three-dimensional tactical principle of “kick from a distance, strike up close, and grapple at close quarters.” It places extremely high demands on athletes’ strength, speed, neuromuscular coordination, and tactical adaptability. During competition, athletes engage in full-contact sparring on a padded ring, aiming to score points by landing effective strikes or executing takedowns. In this study, the side kick emerges as a core and critical technique within Sanda [4]. As a core offensive and defensive technique in Sanda, the side kick combines long attack range, explosive power, and broad control coverage. It serves as a critical means for scoring, counter-defense, and spatial control during combat, with its technical quality directly influencing match outcomes [5]. Its technical quality directly impacts scoring efficiency and defensive success rates during combat [6]. Executing this movement requires coordinated participation of multiple muscle groups, encompassing lower-limb push-off muscles, core stabilizers, and trunk modulator muscles. It involves a sequential process of “push-off-rotation-extension-power generation,” demanding highly synchronized neuromuscular control [7]. Technical variations in the side kick among athletes of different skill levels fundamentally reflect differences in neuromuscular control strategies and muscle coordination efficiency. Intermuscular coherence serves as a key metric for quantifying this core distinction.

By analyzing the synchrony of electrical signals across different muscles, intermuscular coherence reflects the central nervous system’s level of coordinated regulation over multiple muscles, effectively revealing the neural coupling mechanisms between muscles [8]. Existing research has confirmed that intermuscular coherence can distinguish technical proficiency across different sports disciplines, with high-level athletes typically exhibiting superior intermuscular synchrony and coordination efficiency [9,10,11]. Furthermore, coherence holds specific physiological significance across the α/β/γ frequency bands; the β band correlates with movement control and muscle contraction synchrony, while the γ band relates to fine motor regulation and proprioceptive integration, providing support for multidimensional analysis of side kick technique variations [12].

With the rapid advancement of biomechanics and motion sensing technologies, multimodal sensor systems have become essential tools for evaluating movement techniques and revealing performance differences among athletes at various skill levels. Particularly in competitive sports, traditional subjective evaluation methods often struggle to capture subtle variations in movement execution. However, synchronized analysis using sensors such as surface electromyography (sEMG), inertial measurement units (IMUs), and high-speed cameras enables objective quantification of multidimensional metrics including movement dynamics, muscle activation patterns, and neuromuscular coordination.

As one of the core offensive and defensive techniques in Sanda, the execution quality of the side kick directly impacts scoring efficiency and tactical effectiveness during competition. This technique requires athletes to complete a chained sequence—“push off, rotate, extend the knee, and generate force”—within an extremely short timeframe. It involves highly coordinated multi-muscle groups across the upper limbs, lower limbs, and trunk, placing exceptionally high demands on neuromuscular control capabilities. However, current biomechanical research on Sanda side kicks remains limited, particularly lacking empirical studies that systematically reveal technical differences among athletes of varying skill levels from the perspective of intermuscular coordination (e.g., coherence). Existing research predominantly focuses on descriptive analysis or single electromyographic indicators, failing to deeply explore the central nervous system’s role in synchronizing multiple muscle groups. Therefore, this study employs synchronized sensor-based collection of electromyography (EMG) and kinematic data, focusing on the coherence characteristics of key muscle pairs during the side kick movement. It aims to fill this research gap by analyzing the underlying causes of technical proficiency at the neuromuscular coordination level. The findings not only provide a theoretical basis for the scientific training of Sanda side kicks but also establish an objective, quantitative evaluation system for technical assessment and athlete selection in martial arts and combat sports.

This study aims to clarify differences in intermuscular coherence of core muscles across key frequency bands during side kick movements among male Sanda athletes of varying skill levels, thereby revealing the association between neuromuscular coordination mechanisms and technical proficiency.

The findings are expected to enrich neuromuscular control research on Sanda-specific techniques, establish evaluation standards for intermuscular coherence in side kicks, and provide methodological references for quantitative technical analysis in combat sports.

## 2. Research Subjects and Methods

### 2.1. Research Subjects

To ensure statistical power and reliability, this study conducted a prior sample size calculation during the experimental design phase. Using G*Power 3.1 software, based on a moderate effect size (Cohen’s d = 0.8) for electromyographic coherence effects in similar movements from existing research, a two-tailed independent samples *t*-test was conducted with a significance level of α = 0.05 and statistical power = 0.80. Calculations indicated that at least 17 subjects per group were required to achieve sufficient statistical power. Accounting for potential invalid data or dropouts, the final sample size was set at 18 subjects per group, totaling 36 male Sanda athletes. This satisfied the sample size requirements and provided a robust foundation for subsequent intergroup difference analyses. This study included 36 male Sanda athletes, divided into two groups based on athletic level and training duration: the elite athlete group (*n* = 18) and the general athlete group (*n* = 18). Inclusion criteria for the elite athlete group were national first-class or higher Sanda athletes with five or more years of training experience and/or top three finishers in national competitions. The inclusion criteria for the general athlete group were no officially recognized athletic ranking and less than three years of training experience. All participants had no history of musculoskeletal injuries, and their dominant leg was the right leg (to ensure consistency in movements). To standardize movement patterns, all subjects were determined to have a dominant right leg through a combination of functional movement testing and self-reporting; subjects were instructed to stand naturally and perform one vertical jump in place without arm swinging, using each leg, with jump height and movement coordination recorded. They were also asked which leg they habitually used for daily kicking actions such as kicking objects or playing soccer. All participants self-reported and demonstrated superiority of the right leg in both force output and movement control. Furthermore, in the jump test, the height achieved with the dominant right leg exceeded that of the right leg by an average of over 5%. Basic participant information is presented in Table 1, with data expressed as mean ± standard deviation. All experimental procedures in this study strictly adhered to the ethical guidelines of the Declaration of Helsinki and were reviewed and approved by the Ethics Review Committee of Beijing Sport University. All participants fully understood the research objectives, procedures, and potential risks prior to participation and signed written informed consent forms. Throughout the experimental process, researchers consistently prioritized safety, ensuring all testing movements were performed under professional supervision to prevent sports injuries. Participants retained the right to withdraw from the experiment at any time without facing any consequences.

### 2.2. Methods

#### 2.2.1. Experimental Equipment

(1) Noraxon (USA Inc., Scottsdale, AZ, USA) Wireless Surface Electromyography: Surface electromyography signals were acquired using the American Noraxon wireless surface EMG system. The system was configured for 16-channel synchronous recording mode, with a sampling frequency set at 2000 Hz and a system bandwidth of 10–500 Hz. Electrode placement strictly adhered to the standard procedures outlined in the SENIAM international guidelines for surface EMG testing to minimize cross-interference and ensure signal quality.
(2) High-Speed Camera: A 200 Hz high-speed camera system was employed. Spatial calibration was performed using a 12-point 3D calibration frame, ensuring a reprojection error of less than 0.3 mm to meet the precision requirements for subsequent analysis. As shown in the Supplementary Materials.
(3) Other Equipment: Standardized Sanda ring, punching bags, etc.

#### 2.2.2. Experimental Procedure

Prior to formal testing, participants underwent a 15 min standardized warm-up routine comprising dynamic stretching, joint mobilization, and submaximal side kick exercises to familiarize themselves with the experimental protocol and movement requirements. The experiment was conducted in a martial arts training facility with indoor temperatures maintained between 22 and 25 °C to minimize environmental interference with electromyographic signals. Participants wore athletic shorts provided uniformly by the experimenters to expose major lower-body muscle groups for electrode placement. The formal experimental testing comprised a maximum voluntary contraction test and a technical proficiency assessment.

#### 2.2.3. Test Actions

The side kick is an explosive kicking technique requiring full-body coordination. Its core biomechanical process unfolds as follows: It begins with a powerful push-off from the supporting leg (typically the rear leg), providing initial momentum and stability. Simultaneously, the pelvis drives rapid trunk rotation, transferring force to the swinging side. The swinging leg (usually the front leg) sequentially performs hip flexion and knee lift followed by a rapid knee extension, propelling the lower leg along a straight, lateral–anterior trajectory to strike the target with either the ball or heel of the foot. The upper limbs coordinate their swing to maintain dynamic balance. This movement demands a stable supporting leg, rigid core, and a swift, direct force application from the swinging leg to form an efficient kinetic chain. This study’s selection of muscles (15 in total) and division of movement phases (initiation, striking, recovery) are both based on the aforementioned biomechanical characteristics.

(1) Maximum Voluntary Contraction Test: Conducted prior to formal testing, each target muscle performs 3 maximum voluntary contractions, with the highest value used for electromyographic signal normalization.
(2) Side Kick Technique Test: On a standard Sanda ring, 6 side kicks are performed at maximum speed targeting a sandbag positioned at the subject’s chest/rib height. A total of 60 s are allowed between each kick to prevent muscle fatigue accumulation from affecting data quality.

#### 2.2.4. Testing Muscle Selection

**Figure 1:**
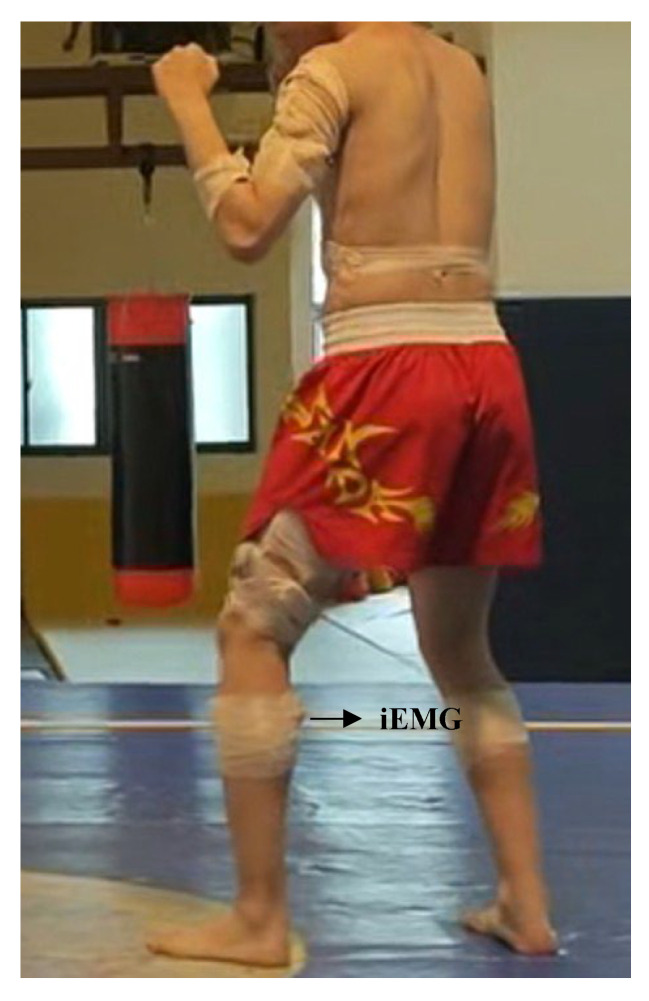
Experimental test site.

Based on the biomechanical characteristics of the side kick movement and prior research foundations, this study selected 15 muscles closely associated with the movement for surface electromyography (sEMG) signal acquisition. The selected muscles included the following: brachioradialis (BR), biceps brachii (BB), triceps brachii (TB), anterior deltoid (AD), external oblique (EO), gluteus maximus (GM), gluteus medius (GMed), biceps femoris (BF), rectus femoris (RF), vastus lateralis (VL), tibialis anterior (TA), and gastrocnemius medial head (GAS), as well as the right tibialis anterior (TAR), right gluteus maximus (GMR), and right rectus femoris (RFR). The aforementioned muscles encompass the primary agonist and synergist muscle groups involved in key movement components during the side kick: trunk stabilization, hip flexion and extension, knee extension, and ankle flexion/extension. This selection comprehensively reflects the neuromuscular activation pattern and bilateral coordination characteristics of this technical movement. See Figure 1 for the experimental test site.

### 2.3. Data Acquisition

EMG signals and kinematic data from 15 target muscles were simultaneously acquired using the Noraxon wireless surface EMG system (2000 Hz) synchronized with a 200 Hz high-speed camera. Electrode placement adhered to SENIAM standards. The side kick motion was divided into three consecutive phases based on biomechanical characteristics: initiation phase, kicking phase, and recovery phase. The acquired EMG signals were subsequently analyzed using coherence analysis and electromyographic indices.

### 2.4. Data Processing

All electromyography and kinematic data collected in this study were processed using specialized computational platforms. The primary tools employed included MATLAB (version 2022a), R (version 4.2.0), and custom Python 3.15scripts. The specific analysis workflow is as follows.

#### 2.4.1. Data Extraction and Preprocessing

Based on synchronized 200 Hz high-speed camera footage and 2000 Hz surface electromyography (EMG) system signals, this study first segmented the complete side kick motion using the two-dimensional motion trajectories captured by the camera (focusing on hip, knee, and ankle joint angles and linear velocities of the swinging leg). According to technical characteristics, the motion cycle was divided into three consecutive phases: initiation phase, kicking phase, and recovery phase. High-frequency EMG data corresponding to each phase was extracted via synchronized time signals for subsequent analysis.

The preprocessing workflow for raw EMG signals included the following: first, applying a fourth-order Butterworth bandpass filter (20–400 Hz) to eliminate power-line interference and motion artifacts; followed by full-wave rectification; and finally smoothing the signal through a fourth-order 20 Hz low-pass filter. To eliminate inter-subject variability (e.g., effects of subcutaneous fat thickness and electrode placement on raw signal amplitude), all EMG data were normalized to each subject’s maximum voluntary contraction (MVC) value. Specifically, the amplitude of each sampled EMG signal point was divided by the subject’s corresponding muscle MVC amplitude, expressing signal amplitude as a dimensionless relative value relative to personal maximum contraction capacity. Subsequently extracted metrics such as root mean square (RMS) and integrated electromyography (iEMG) values were calculated based on these normalized signals [13]. Consequently, while their units were formally retained, they actually represented relative activation levels relative to the individual’s MVC, enabling direct intergroup comparisons.

Based on synchronized signals from a 200 Hz high-speed camera and a 2000 Hz sampling rate surface electromyography system, the complete action cycle—from the starting posture through execution of the side kick to the return to fighting stance—was first defined according to the kinetic characteristics of the side kick movement. High-frequency electromyography data for the corresponding time intervals was extracted via synchronized time signals for subsequent analysis.

The preprocessing workflow for raw EMG signals included the following: first applying a fourth-order Butterworth bandpass filter (20–400 Hz) to eliminate power-line interference and motion artifacts; followed by full-wave rectification; and finally smoothing the signal through a fourth-order, 20 Hz low-pass filter. To account for inter-subject variability, all EMG data were normalized relative to each participant’s maximum voluntary contraction value.

#### 2.4.2. Electromyographic Feature Extraction

(1) Root Mean Square (RMS) Amplitude

The root mean square value of the electromyographic signal is calculated using the sliding window method, with a window length of 100 ms and a step size of 50 ms. The calculation formula is as follows:

$$RMS=\sqrt{\frac{1}{N}\sum_{i=1}^{N}{x_{i}}^{2}}$$

where xi represents the electromyographic signal value, and N denotes the number of sampling points within the window. Prior to calculation, the signal undergoes 20 Hz high-pass filtering to eliminate baseline drift.

(2) Integral Electromyography (iEMG)

Numerical integration is performed on the full-wave rectified electromyographic signal during each phase of the side kick leg movement. The calculation formula is:

$$iEMG=\int_{t_{1}}^{t_{2}}|x(t)|dt$$

where t1 and t2 represent the start and end times of each movement phase, respectively. To eliminate differences in movement duration, all results were normalized by dividing them by the phase duration.

#### 2.4.3. Calculation of Intramuscular Time-Frequency Coherence

Based on the Short-Time Fourier Transform (STFT), muscle inter-temporal frequency coherence (TFC) analysis was implemented using a custom-developed Python program. A 20–400 Hz bandpass filter was applied to eliminate low-frequency motion artifacts and high-frequency noise, followed by full-wave rectification processing. During the spectral analysis phase, the signal was segmented using a Hamming window function (window length: 200 samples; overlap: 75%) to compute the mutual power spectral density between muscle pairs and the self-power spectral density for each muscle. To enhance data stability, the results were smoothed using a two-dimensional convolution kernel.

The time-frequency coherence values were ultimately calculated using the following formula:

Cxy(l,f)=|p^xy[l,f]⊗v[t]|2{|p^xx∧[l,f]|2⊗v[t]}{|p^yy[l,f]|2⊗v[t]}

The symbol ⊗ denotes the convolution operation. TFC is normalized to the range 0–1, with values closer to 1 indicating stronger coherence.

#### 2.4.4. Statistical Analysis

This study employed SPSS 26.0 software for statistical analysis. Outliers were first identified and excluded using box plots, and data normality was assessed via the Shapiro–Wilk test. Subsequently, normally distributed data underwent independent samples *t*-tests for intergroup comparisons (professional group vs. amateur group), while non-normally distributed data utilized the Mann–Whitney U test. All results are presented as mean ± standard deviation (Mean ± SD), with statistical significance set at *p* < 0.05.

## 3. Results

### 3.1. Electromyographic Characteristic Indicators

During the side kick movement, the professional group demonstrated significantly higher values in RF (RMS and iEMG both P < 0.001), VL (RMS P = 0.002, iEMG P = 0.001), AD (RMS P = 0.008, iEMG P = 0.007), EO (both P < 0.001), TAR (RMS P = 0.039, iEMG P = 0.037), GMR (RMS P = 0.013, iEMG P = 0.012), RFR (RMS P = 0.010, iEMG P = 0.009), and BB (RMS P = 0.021, iEMG P = 0.019), which were significantly higher in the professional group than in the amateur group. However, no significant differences were observed between the two groups for BR, TB, GM, GMed, BF, TA, or GAS, as shown in Table 2.

### 3.2. Muscle Interconnectivity

#### 3.2.1. Differences in Coordination Between Upper Limbs and Swing Leg

**Figure 2:**
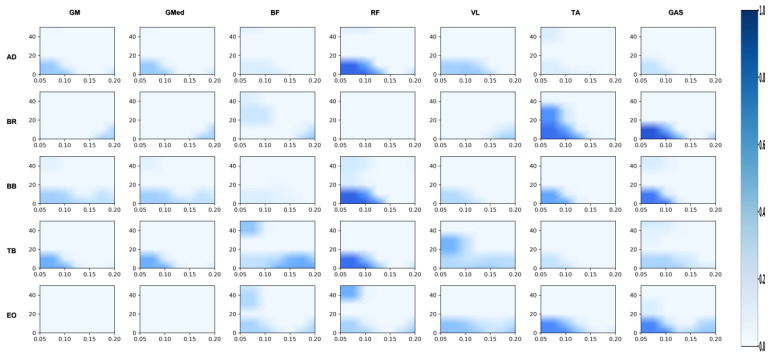
Amateur group: analysis of the correlation between the swing leg and the upper limb/trunk. Note: This heatmap illustrates the strength of time-frequency coherence between upper limb/trunk muscles and swing leg muscles during side kicks performed by amateur athletes. The horizontal axis represents time (s), while the vertical axis represents frequency (Hz). Colors range from light to dark, indicating coherence values from low to high.

**Figure 3:**
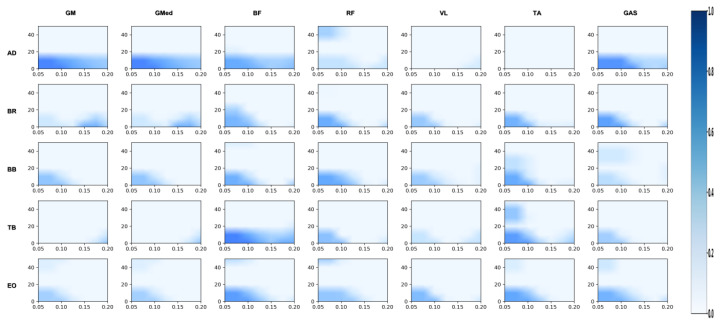
Professional group: coherence analysis of swing leg and upper limb/trunk. Note: This heatmap displays the time-frequency coherence strength between upper limb/trunk muscles and swing leg muscles during side kicks performed by professional athletes. The horizontal axis represents time (s), and the vertical axis represents frequency (Hz). Colors range from light to dark, indicating coherence values from low to high.

**Figure 4:**
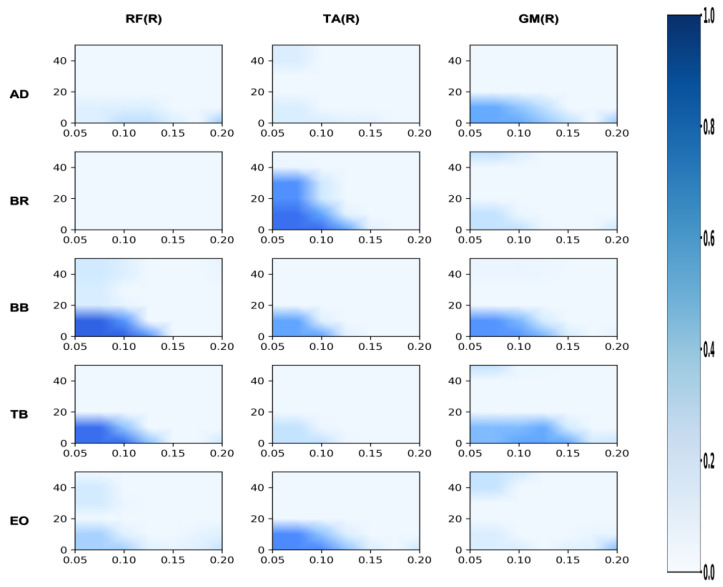
Amateur group: correlation analysis of support leg and upper limb/trunk. Note: This heatmap displays the time-frequency coherence strength between upper limb/trunk muscles and support leg muscles during the side kick movement for amateur athletes. The horizontal axis represents time (s), and the vertical axis represents frequency (Hz). Colors range from light to dark, indicating coherence values from low to high.

**Figure 5:**
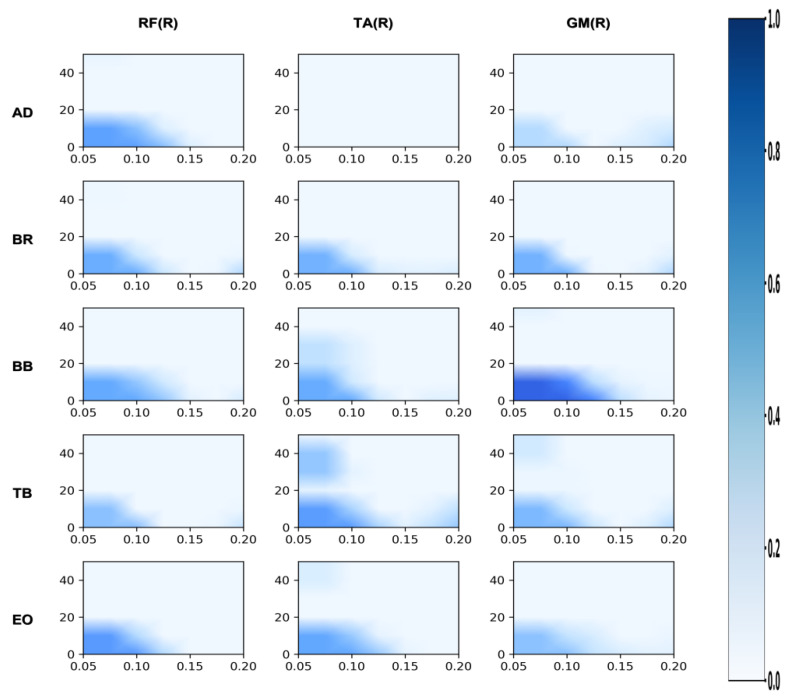
Professional group: correlation analysis of support leg and upper limb/trunk. Note: This heatmap displays the strength of time-frequency coherence between upper limb/trunk muscles and supporting leg muscles during side kicks performed by professional athletes. The horizontal axis represents time (s), and the vertical axis represents frequency (Hz). Colors range from light to dark, indicating coherence values from low to high.

**Figure 6:**
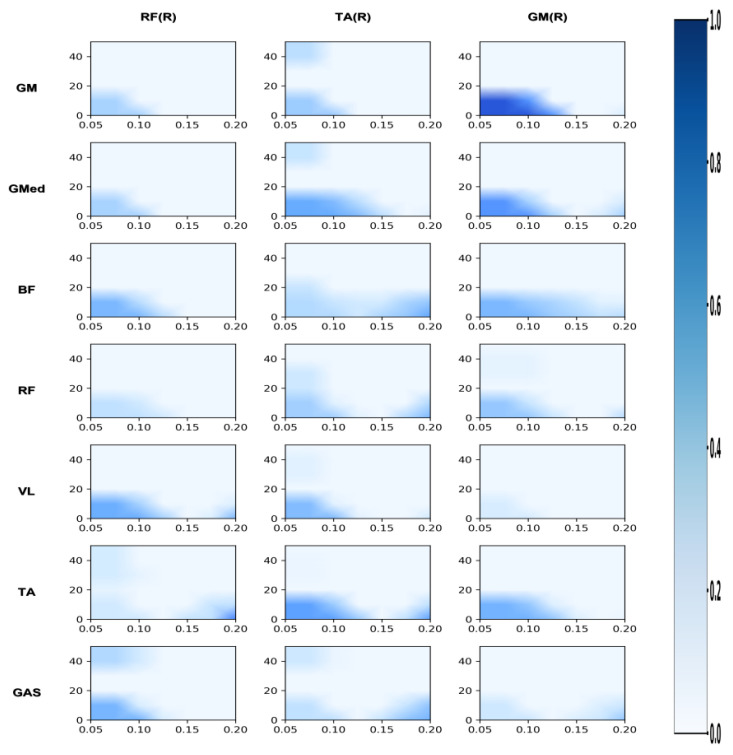
Amateur division: correlation analysis of support leg and swing leg. Note: This heatmap displays the strength of time-frequency coherence between muscles of the support leg and swing leg during the side kick movement performed by amateur athletes. The horizontal axis represents time (s), and the vertical axis represents frequency (Hz). Colors range from light to dark, indicating coherence values from low to high.

**Figure 7:**
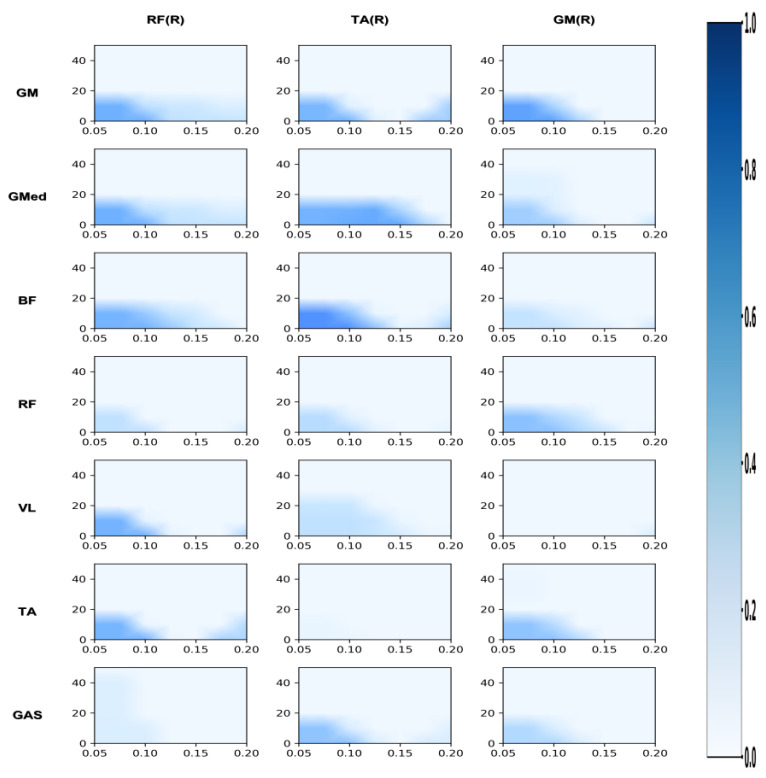
Professional group: coherence analysis of support leg and swing leg. Note: This heatmap displays the strength of time-frequency coherence between muscles of the support leg and swing leg during side kicks performed by professional athletes. The horizontal axis represents time (s), and the vertical axis represents frequency (Hz). Colors range from light to dark, indicating coherence values from low to high.

Figure 2, Figure 3, Figure 4, Figure 5, Figure 6 and Figure 7 reflect the coherence results. In terms of muscle coordination between the upper limbs and swing leg, the professional group demonstrated significant advantages across multiple frequency bands. In the Alpha band (8–15 Hz), the professional group exhibited higher coherence between the anterior deltoid and gluteus medius (AD_GMed), biceps brachii and rectus femoris (BB_RF), biceps brachii and vastus lateralis (BB_VL), external oblique and gastrocnemius (EO_GAS), and external oblique and rectus femoris (EO_RF), which were significantly higher than those of the amateur group (*p* < 0.05). The difference in EO_RF reached a highly significant level (*p* < 0.01). In the Beta band (15–30 Hz), the coherence values for AD_GMed, triceps brachii and tibialis anterior (TB_TA), and BB_VL also significantly outperformed those of the amateur group (*p* < 0.05). In the Gamma band (30–50 Hz), the professional group also exhibited significantly higher coherence values for the brachioradialis and biceps femoris (BR_BF) and the external oblique and gluteus medius (EO_GMed) compared to the amateur group (*p* < 0.05).

#### 3.2.2. Differences in Coordination Between the Support Leg and the Swing Leg

In terms of coordination between the supporting leg and the swinging leg, the professional group also demonstrated a significant advantage. In the Alpha band, the professional group exhibited significantly higher coherence values than the amateur group for gluteus medius with tibialis anterior (GMed_TAR), right rectus femoris with biceps femoris (RFR_BF), and right rectus femoris with tibialis anterior (RFR_TA) (*p* < 0.05), with the difference in RFR_BF reaching a highly significant level (*p* < 0.01). In the Beta band, the coherence values for GMed_TAR and RFR_BF were also significantly superior to those of the amateur group (*p* < 0.05), with RFR_BF again exhibiting a highly significant difference (*p* < 0.01).

#### 3.2.3. Differences in Coordination Between Upper Limbs and Supporting Legs

**Figure 8:**
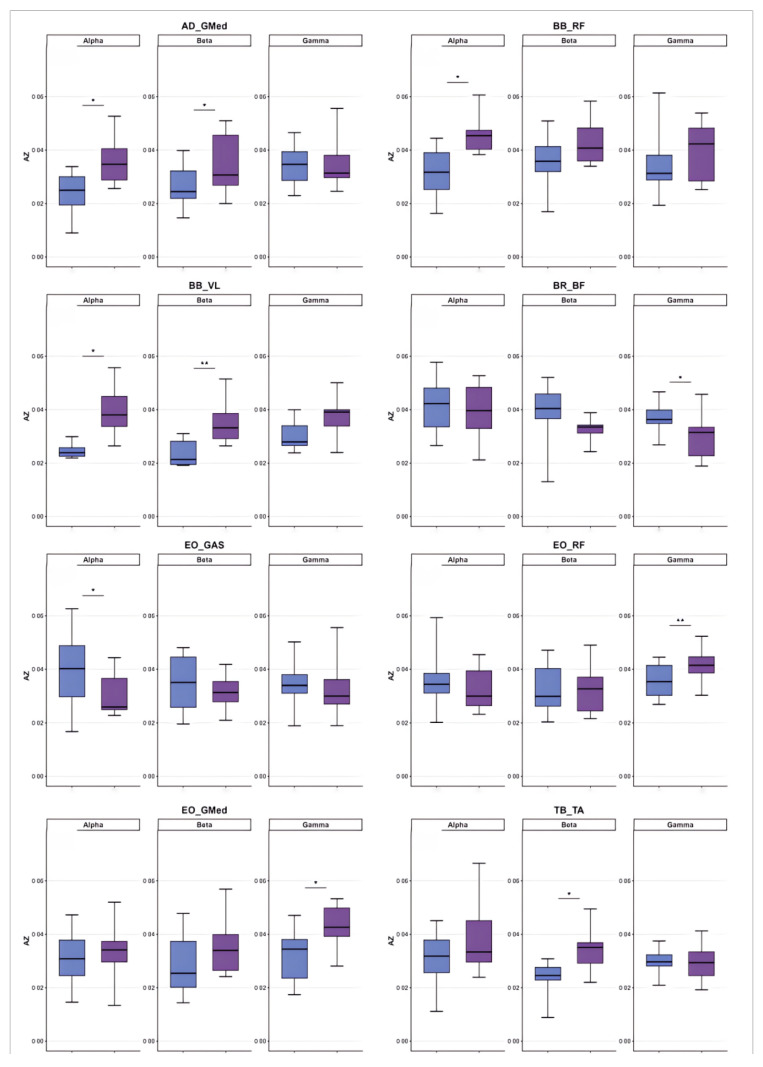
Comparative analysis of coordination between swing leg and upper limb/trunk muscle groups. Note: purple: professional group; blue: amateur group.

**Figure 9:**
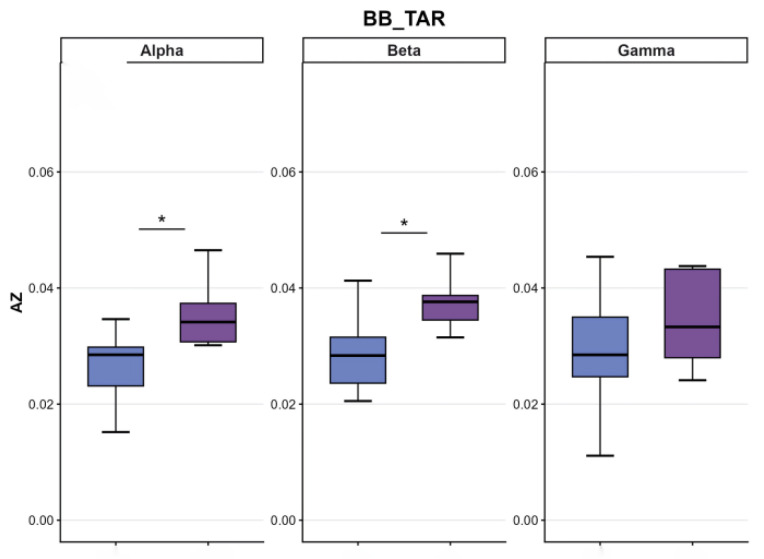
Comparative analysis of coordination between support legs and upper limb/trunk muscle groups. Note: purple: professional group; blue: amateur group.

**Figure 10:**
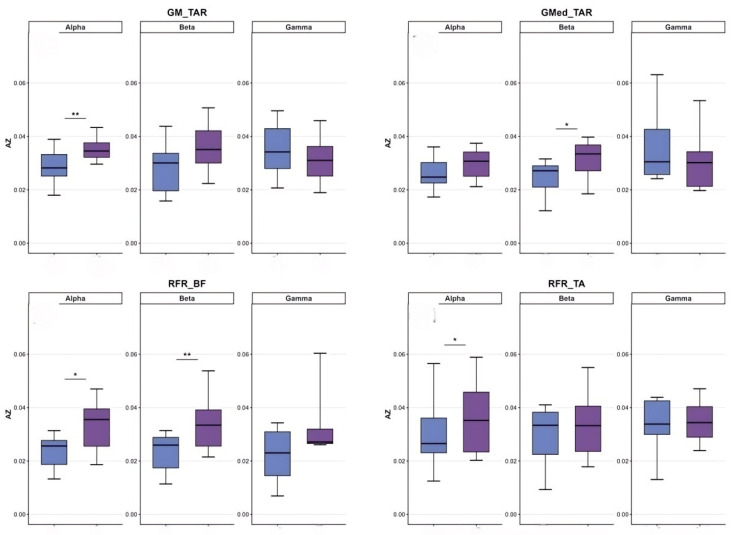
Comparative analysis of muscle coordination between support and swinging legs. Note: purple: professional group; blue: amateur group.

In terms of upper limb and supporting leg coordination, the professional group demonstrated a significant advantage in the biceps brachii and right tibialis anterior muscle pair (BB_TAR). In the Alpha band, the BB_TAR coherence values of the professional group were significantly higher than those of the amateur group (*p* < 0.05). In the Beta band, BB_TAR coherence values were also significantly superior to those of the amateur group (*p* < 0.05), as shown in Table 3 and in Figure 8, Figure 9 and Figure 10.

## 4. Discussion

By comparing the intermuscular coherence and electromyographic characteristics of side kick movements between professional and amateur Sanda athletes, this study identified significant differences in neuromuscular coordination patterns between the two groups. These differences were concentrated in key muscle pairs and specific frequency bands, closely aligning with the biomechanical demands of the side kick sequence: ground push-off, rotation, extension, and force generation.

Compared to the amateur group, the professional group exhibited significantly higher intermuscular coherence in the following muscle pairs: external oblique–rectus femoris in the α band (8–15 Hz); biceps brachii–rectus femoris in the β band (15–30 Hz); anterior deltoid–gluteus medius in the β band; triceps brachii–tibialis anterior in the β band; external oblique–gluteus medius in the γ band (30–50 Hz); and brachioradialis–biceps femoris in the γ band. Notably, the external oblique–gluteus medius and brachioradialis–biceps femoris pairs showed extremely significant differences in the α band. The external oblique–rectus femoris pair exhibited a highly significant difference in the alpha band. This indicates that elite athletes exhibit tighter neural coupling between trunk stabilizers and lower limb extensors during the side kick initiation and striking phases. They can provide stable support for lower limb swing through coordinated upper limb and trunk force generation, achieving efficient force transmission. In contrast, the amateur group exhibited lower intermuscular coherence in these muscle pairs, reflecting disorganized coordination between trunk and lower limb muscles. This resulted in increased energy loss during force generation and difficulty in forming a unified force, consistent with the commonly observed characteristics among secondary-level athletes, such as uneven muscle activation and insufficient involvement of the lumbar and abdominal muscles [14].

The professional group demonstrated significantly superior muscle pair coherence compared to the amateur group in the α band (right rectus femoris–biceps femoris; right rectus femoris–tibialis anterior) and β band (gluteus medius–right tibialis anterior; right rectus femoris–biceps femoris). Notably, the right rectus femoris–biceps femoris pair exhibited extremely significant differences across both α and β bands. Highly efficient coordination between the supporting and swinging legs is central to ensuring stability and force transmission efficiency in the side kick movement. Elite athletes achieve precise synchronization between the supporting leg’s stable foundation and the swinging leg’s explosive power through synchronized regulation of bilateral lower limb muscles. Amateur athletes, however, often exhibit disrupted muscle coordination rhythms, leading to issues like unstable support legs and movement imbalance. This contradicts the core requirements of the side kick technique—stable support and rapid swing—and aligns with the common technical deficiencies observed in second-tier athletes, including hip and knee joint angles, movement prioritization, and the need for improved lower limb coordination and stability [15].

The professional group exhibited significantly higher biceps brachii–right tibialis anterior coherence in the α and β frequency bands compared to the amateur group. This indicates that elite athletes optimize balance control and force transmission in the supporting leg through auxiliary regulation by upper limb muscles, closely related to the role of upper limb swing in adjusting the body’s center of gravity during the side kick. EMG characteristics revealed that the professional group exhibited higher root mean square amplitude and integral EMG values in most core muscles compared to the amateur group. Key muscles such as the tibialis anterior and vastus lateralis demonstrated activation intensities better aligned with technical demands. In contrast, the amateur group exhibited disordered muscle activation sequences and insufficient core muscle engagement. This aligns with findings from EMG studies of high-level Sanda athletes performing side kicks, which also indicated more rational muscle activation timing [16].

The essence of intermuscular coherence lies in the central nervous system’s ability to synchronously regulate multiple muscle groups. Differences in neural control strategies among athletes of varying skill levels are the core reason for this differentiation in coherence. Through long-term specialized training, elite athletes develop highly efficient neural circuit regulation patterns. The integration efficiency of the motor cortex and cerebellum in coordinating muscle synergies significantly improves, enabling precise regulation of the activation timing and synchrony of trunk, upper limb, and lower limb muscles according to the technical demands of different phases of the side kick. This aligns with findings by Dai Ming et al., who observed that elite athletes exhibit superior central nervous system coupling control over multiple muscles, resulting in significantly higher intermuscular coherence compared to novices [17].

β-band coherence is closely associated with motor control and muscle contraction synchrony. The high coherence observed in key muscle pairs within this band among the professional group reflects more precise synchronous regulation of muscle contractions by their motor cortex. This aligns with Santos et al.’s perspective that enhanced β-band coherence signifies mature neuromuscular coordination [18]. The γ band correlates with fine motor regulation and proprioceptive integration. The high coherence observed in specific muscle pairs within this band among the professional group suggests superior proprioceptive and fine motor control capabilities. In contrast, the amateur group, with shorter training durations, exhibited immature neural control strategies and disordered muscle activation timing, resulting in low intermuscular neural coupling and poor coherence performance.

The intensity, duration, and specificity of specialized training directly influence intermuscular coherence levels. Professional athletes averaged 7.8 ± 2.5 years of training experience. Long-term specialized side kick training fostered stable muscle memory, with multi-muscle group coordination patterns optimized through repeated reinforcement. Research indicates that during long-term athletic training, calcium ion signaling in astrocytes can remodel synaptic connections by maintaining synaptic potentiation. This ensures stability in central nervous system control over muscle movement, thereby enhancing athletic performance [19]. Concurrently, prolonged training remodels synaptic connections in the spinal cord and central nervous system, optimizing the central nervous system’s coordinated control strategy for lower limb muscles during the start phase [20].

The amateur group had only 1.8 ± 0.9 years of training experience, lacked systematic coordinated force training, exhibited illogical muscle engagement patterns, and struggled to develop efficient muscle coordination. Moreover, the professional group demonstrated a deeper understanding of the counter-kicking technique, precisely controlling the rhythmic coordination of muscles across different phases. In contrast, the amateur group frequently exhibited technical irregularities such as unstable supporting legs and insufficient trunk rotation, further exacerbating differences in intermuscular coherence. This aligns with existing research findings that high-level martial arts athletes exhibit more coordinated muscle cooperation during movement execution, resulting in overall greater energy efficiency [21].

Foundational physical capabilities like muscular strength, core stability, and proprioception provide essential support for neuromuscular coordination. Professional athletes’ long-term training has cultivated superior lower-body strength and core stability, establishing a robust biomechanical foundation for coordinated muscle contraction and minimizing disruptive compensatory force patterns. Concurrently, specialized training enhances the professional group’s proprioceptive acuity, enabling real-time perception of postural changes during movement and prompt adjustments to muscle activation to maintain intermuscular coordination efficiency [22].

Amateur athletes exhibit relatively weaker muscle strength and proprioception, making them prone to postural instability during exertion. This instability triggers compensatory muscle activation, disrupting normal synergistic patterns and manifesting as reduced intermuscular coherence. This finding confirms that fundamental physical capabilities are prerequisites for efficient neuromuscular coordination, consistent with conclusions that inadequate core stability leads to movement imbalance and strength loss [23,24]. The efficacy of core stability training in enhancing muscle coordination efficiency has been demonstrated across multiple sports studies and rehabilitation contexts, as it optimizes neuromuscular control patterns to improve synchronized multi-muscle activation [25,26].

This study holds significant theoretical and practical value. Theoretically, it systematically reveals intermuscular coherence differences in side kick movements among Sanda athletes of varying skill levels, clarifying the coherence characteristics of key muscle pairs in the β and γ frequency bands. It establishes quantitative evaluation standards for intermuscular coherence in side kicks, confirming its utility as an objective indicator of skill level in zone-scoring Sanda, thereby expanding the assessment framework for Sanda athletes’ technical proficiency. β, and γ frequency bands for key muscle pairs establishes quantitative evaluation standards for intermuscular coherence in side kicks, confirms its utility as an objective indicator for differentiating Sanda athletes’ technical proficiency, expands the application of electromyographic signals in combat sports, addresses limitations of traditional subjective assessments, enriches the neuromuscular control research framework for Sanda-specific techniques, and deepens understanding of the neuromuscular control mechanisms underlying side kicks. In practice, this provides precise optimization directions for specialized Sanda side kick training. For amateur athletes exhibiting coherence deficits in key muscle pairs within specific frequency bands, targeted interventions—such as upper-limb–trunk–lower-limb coordinated force generation exercises, movement decomposition and integration training, and EMG feedback training—can enhance muscle synchrony and neural control efficiency. Concurrently, intermuscular coherence serves as a quantifiable metric for training effectiveness and athlete selection, enhancing training science and precision while reducing talent identification costs.

Strengths and Limitations: This study’s strength lies in its pioneering integration of multi-sensor synchronous data acquisition with intermuscular time-frequency coherence analysis. It systematically reveals neuromuscular coordination differences in side kick techniques among Sanda athletes of varying skill levels, providing objective and quantifiable new metrics for technical evaluation. However, certain limitations remain: First, the sample size was relatively small and exclusively comprised male athletes, limiting the generalizability of findings to females or broader populations. Second, surface electromyography signals are susceptible to factors such as subcutaneous tissue and electrode placement, and coherence analysis primarily reflects coupling at spinal cord and higher levels, offering limited insight into intraspinal mechanisms. Finally, this cross-sectional study revealed correlations but cannot directly infer causality between long-term training and improved coherence. Future research should expand sample sizes, incorporate multidimensional neuroimaging techniques, and adopt longitudinal intervention designs to further elucidate training-induced neuroplasticity mechanisms.

## 5. Conclusions

This study investigated differences in muscle inter-reaction coherence and electromyographic characteristics during side kicks among male Sanda athletes of varying skill levels through surface electromyography analysis, revealing the core mechanisms underlying skill differentiation. Results indicate that the professional group exhibited significantly higher root mean square (RMS) amplitude and integral electromyography (iEMG) values in most core muscles compared to the amateur group. Key muscle activation intensity better aligned with technical demands, whereas the amateur group demonstrated disordered muscle activation sequences and insufficient core engagement. Regarding intermuscular coherence, the professional group demonstrated significantly superior coordination in key muscle pairs—the upper limb–swinging leg, support leg–swinging leg, and upper limb–support leg—in the α, β, and γ frequency bands compared to the amateur group. Particularly notable coherence differences were observed in muscle pairs such as the external oblique–rectus femoris, right rectus femoris–biceps femoris, and right biceps femoris–right tibialis anterior, which exhibited particularly pronounced differences in coherence, ensuring movement stability and force transmission efficiency.

Intermuscular coherence serves as an objective quantitative indicator for assessing the technical proficiency of side kick in Sanda athletes. The coherence characteristics of key muscle pairs in the α and β frequency bands can be utilized for technical evaluation and training effectiveness assessment. These findings provide precise optimization directions for specialized training of side kick in Sanda and offer methodological support for the quantitative analysis of techniques in combat sports.

## Figures and Tables

**Table 1:** Characteristics of the participants (*n* = 36).

Group	Age (Years)	Height (cm)	Mass (kg)	Training Experience (Years)	Dominant Leg
Professional	22.5 ± 3.1	176.5 ± 6.2	70.2 ± 18.3	7.8 ± 2.5	Right
Amateur	21.8 ± 1.9	175.8 ± 5.9	68.9 ± 12.8	1.8± 0.9	Right

**Table 2:** Comparison of electromyographic parameters for side kick movements between professional and amateur groups (mean ± SD).

Muscle Name	Grouping	RMS	*p*-Value	iEMG	*p*-Value
BR	Professional	0.085 ± 0.024	0.087	122.4 ± 34.9	0.091
	Amateur	0.072 ± 0.029		103.7 ± 41.4	
BB	Professional	0.124 ± 0.035	0.021	178.5 ± 50.6	0.019
	Amateur	0.098 ± 0.040		141.1 ± 57.4	
TB	Professional	0.135 ± 0.038	0.522	194.4 ± 55.2	0.515
	Amateur	0.142 ± 0.045		204.5 ± 64.5	
AD	Professional	0.187 ± 0.050	0.008	269.3 ± 71.4	0.007
	Amateur	0.145 ± 0.056		208.8 ± 80.6	
EO	Professional	0.254 ± 0.061	<0.001	365.8 ± 87.5	<0.001
	Amateur	0.182 ± 0.067		262.1 ± 96.8	
GM	Professional	0.231 ± 0.058	0.452	332.6 ± 82.9	0.467
	Amateur	0.218 ± 0.072		313.9 ± 103.7	
GMed	Professional	0.165 ± 0.045	0.054	237.6 ± 64.5	0.051
	Amateur	0.132 ± 0.051		190.1 ± 73.8	
BF	Professional	0.156 ± 0.043	0.334	224.6 ± 62.2	0.310
	Amateur	0.168 ± 0.056		241.9 ± 80.6	
RF	Professional	0.298 ± 0.072	<0.001	429.1 ± 103.7	<0.001
	Amateur	0.214 ± 0.077		308.2 ± 110.6	
VL	Professional	0.267 ± 0.066	0.002	384.5 ± 94.4	0.001
	Amateur	0.198 ± 0.070		285.1 ± 101.4	
TA	Professional	0.178 ± 0.048	0.064	256.3 ± 69.1	0.061
	Amateur	0.145 ± 0.056		208.8 ± 80.6	
GAS	Professional	0.195 ± 0.053	0.075	280.8 ± 76.0	0.071
	Amateur	0.162 ± 0.061		233.3 ± 87.5	
TAR	Professional	0.165 ± 0.045	0.039	237.6 ± 64.5	0.037
	Amateur	0.128 ± 0.050		184.3 ± 71.4	
GMR	Professional	0.198 ± 0.051	0.013	285.1 ± 73.8	0.012
	Amateur	0.154 ± 0.058		221.8 ± 82.9	
RFR	Professional	0.187 ± 0.050	0.010	269.3 ± 71.4	0.009
	Amateur	0.142 ± 0.054		204.5 ± 77.4	

Brachioradialis (BR), biceps brachii (BB), triceps brachii (TB), anterior deltoid (AD), external oblique (EO), gluteus maximus (GM), gluteus medius (GMed), biceps femoris (BF), rectus femoris (RF), vastus lateralis (VL), tibialis anterior (TA), gastrocnemius medial head (GAS), the right tibialis anterior (TAR), right gluteus maximus (GMR), and right rectus femoris (RFR).

**Table 3:** Comparison of muscle inter-correlation in side kick movements between professional and amateur groups (mean ± SD).

Comparison	Parameters	Frequency Band	Amateur	Professional
Upper limbs vs. swinging legs	AD_GMed	Alpha	0.032 ± 0.011	0.035 ± 0.01 *
	AD_GMed	Beta	0.028 ± 0.01	0.034 ± 0.009 *
	AD_GMed	Gamma	0.028 ± 0.01	0.031 ± 0.009
	BB_RF	Alpha	0.031 ± 0.011	0.039 ± 0.012 *
	BB_RF	Beta	0.034 ± 0.013	0.039 ± 0.01
	BB_RF	Gamma	0.037 ± 0.008	0.038 ± 0.009
	TB_TA	Alpha	0.03 ± 0.01	0.038 ± 0.014
	TB_TA	Beta	0.023 ± 0.006	0.034 ± 0.008 *
	TB_TA	Gamma	0.029 ± 0.004	0.029 ± 0.006
	BB_VL	Alpha	0.037 ± 0.011	0.037 ± 0.004 *
	BB_VL	Beta	0.032 ± 0.008	0.035 ± 0.006 **
	BB_VL	Gamma	0.035 ± 0.010	0.032 ± 0.007
	BR_BF	Alpha	0.039 ± 0.013	0.038 ± 0.013
	BR_BF	Beta	0.033 ± 0.005	0.032 ± 0.012
	BR_BF	Gamma	0.033 ± 0.008	0.036 ± 0.009 *
	EO_GAS	Alpha	0.038 ± 0.010	0.035 ± 0.011 *
	EO_GAS	Beta	0.034 ± 0.007	0.032 ± 0.011
	EO_GAS	Gamma	0.033 ± 0.009	0.035 ± 0.006
	EO_GMed	Alpha	0.029 ± 0.009	0.03 ± 0.005
	EO_GMed	Beta	0.031 ± 0.011	0.03 ± 0.008
	EO_GMed	Gamma	0.03 ± 0.007	0.034 ± 0.009 *
	EO_RF	Alpha	0.032 ± 0.011	0.039 ± 0.012
	EO_RF	Beta	0.033 ± 0.009	0.04 ± 0.008
	EO_RF	Gamma	0.03 ± 0.014	0.034 ± 0.007 **
Supporting leg vs. swing leg	GM_TAR	Alpha	0.031 ± 0.009	0.031 ± 0.006 **
	GM_TAR	Beta	0.032 ± 0.009	0.031 ± 0.006
	GM_TAR	Gamma	0.034 ± 0.008	0.032 ± 0.01
	GMed_TAR	Alpha	0.025 ± 0.006	0.029 ± 0.005
	GMed_TAR	Beta	0.024 ± 0.006	0.031 ± 0.006 *
	GMed_TAR	Gamma	0.035 ± 0.012	0.03 ± 0.009
	RFR_BF	Alpha	0.023 ± 0.006	0.033 ± 0.01 *
	RFR_BF	Beta	0.023 ± 0.007	0.033 ± 0.01 **
	RFR_BF	Gamma	0.022 ± 0.01	0.032 ± 0.011
	RFR_TA	Alpha	0.03 ± 0.014	0.036 ± 0.014 *
	RFR_TA	Beta	0.029 ± 0.011	0.033 ± 0.012
	RFR_TA	Gamma	0.033 ± 0.01	0.035 ± 0.008
Upper body vs. supporting leg	BB_TAR	Alpha	0.032 ± 0.009	0.038 ± 0.011 *
	BB_TAR	Beta	0.032 ± 0.005	0.037 ± 0.008 *
	BB_TAR	Gamma	0.028 ± 0.006	0.031 ± 0.009

Note: * or ** indicates a significant difference compared to the amateur group.

## Data Availability

All data are included in the text. For any additional requirements, please contact the corresponding author.

## Supplementary Materials

The following supporting information can be downloaded at: https://www.mdpi.com/article/10.3390/s26020646/s1.
